# Detection of human auditory evoked brain signals with a resilient nonlinear optically pumped magnetometer

**DOI:** 10.1016/j.neuroimage.2020.117497

**Published:** 2021-02-01

**Authors:** Anna U. Kowalczyk, Yulia Bezsudnova, Ole Jensen, Giovanni Barontini

**Affiliations:** a Centre for Human Brain Health, School of Psychology, University of Birmingham, Edgbaston, Birmingham B15 2SA, United Kingdom; b School of Physics and Astronomy, University of Birmingham, Edgbaston, Birmingham B15 2TT, United Kingdom

**Keywords:** Optically pumped magnetometer, Nonlinear magneto-optical rotation, Magnetoencephalography, Auditory evoked response

## Abstract

Optically Pumped Magnetometers (OPMs) have been hailed as the future of human magnetoencephalography, as they enable a level of flexibility and adaptability that cannot be obtained with systems based on superconductors. While OPM sensors are already commercially available, there is plenty of room for further improvements and customization. In this work, we detected auditory evoked brain fields using an OPM based on the nonlinear magneto-optical rotation (NMOR) technique. Our sensor head, containing only optical and non-magnetizable elements, is connected to an external module including all the electronic components, placed outside the magnetically shielded room. The use of the NMOR allowed us to detect the brain signals in non-zero magnetic field environments. In particular, we were able to detect auditory evoked fields in a background field of 70 nT. We benchmarked our sensor with conventional SQUID sensors, showing comparable performance. We further demonstrated that our sensor can be employed to detect modulations of brain oscillations in the alpha band. Our results are a promising stepping-stone towards the realization of resilient OPM-based magnetoencephalography systems that do not require active compensation.

## Highlights

• We have built and tested an Optically Pumped Magnetometer based on non-linear magnetooptical rotation.
• We present a new approach to OPM- based MEG using a modular NOPM sensor.
• Our sensor is resilient to non-zero and non-uniform magnetic field environments and crosstalk free.
• We demonstrate the operation of the NOPM sensor by measuring auditory response and calculating time-frequency representation of power.

## 1 Introduction

Techniques for electrophysiological brain recordings utilizing SQUID-based magnetoencephalography (MEG) have greatly evolved over the last few decades, providing important clinical and cognitive neuroscience insight. In particular, when employing more than than 100 sensors, MEG enables a general approach to localize the measured signals in the brain using source modelling (Hämäläinen et al., 1993). The main disadvantage of SQUID-based MEG systems is that they require the sensors to be immersed in liquid helium; as a result, these systems are costly, and the required thermal insulation reduces the signal-to-noise ratio, as it limits how close the sensors can go to the scalp.

The application of Optically Pumped Magnetometers (OPMs) in human magnetoencephalographic recordings has been hailed as an exciting approach, as it holds the promise of reducing costs while improving the signal-to-noise ratio. Recent work has provided important proofs-of-principle that OPMs can be applied to record human brain activity, ranging from stimulus evoked responses (event-related fields; ERFs) to modulations in neuronal oscillations (Borna, Carter, Goldberg, Colombo, Jau, Berry, McKay, Stephen, Weisend, Schwindt, 2017, Boto, Holmes, Leggett, Roberts, Shah, Meyer, Muñoz, Mullinger, Tierney, Bestmann, Barnes, Bowtell, Brookes, 2018, Iivanainen, Zetter, Parkkonen, 2019, Johnson, Schwindt, Weisend, 2010, Sander, Preusser, Mhaskar, Kitching, Trahms, Knappe, 2012, Sheng, Wan, Yifan, Dou, Guo, Wei, He, Qin, Gao, 2017, Xia, Banranga, Hoffman, Romalis, 2007). One of the most exciting features of OPM sensors is that they can be arranged in arbitrary arrays. This means that they can be adapted to individual head shapes, making them more resilient against head movements, and they can be applied to children (Hill et al., 2019). Recent studies have confirmed that the potential gain in the signal-to-noise ratio featured by the OPMs can improve the accuracy of source modelling (Boto, Bowtell, Krüger, Fromhold, Morris, Meyer, Barnes, Brookes, 2016, Zetter, Iivanainen, Stenroos, Parkkonen, 2018). This will allow one to infer with greater precision where in the brain the measured signals are generated.

So far, the recordings in humans have mainly been implemented with so-called ”zero-field” magnetometers, which comprise the vast majority of commercially available sensors. Since zero-field sensors often exploit the spin-exchange relaxation-free (SERF) effect, they are usually also called ”SERF sensors”. These have typical sensitivities of 10–15 fT$/\sqrt{\text{Hz}}$ (Osborne et al., 2018). One of the main drawbacks of zero-field sensors is that they can be affected by cross-talk when multiple sensors are employed. The coils embedded in the sensor, used for the modulation of the magnetic field and/or for DC field zeroing, can indeed affect the sensitivity and the accuracy of multi-sensor arrays (Osborne, Orton, Alem, Shah, 2018, Tierney, Holmes, Mellor, López, Roberts, Hill, Boto, Leggett, Shah, Brookes, Bowtell, Barnes, 2019). The impact of these issues can be compensated or reduced by using high-performance shielded rooms or advanced and adaptive external field nulling coils (Holmes, Tierney, Leggett, Boto, Mellor, Roberts, Hill, Shah, Barnes, Brookes, Bowtell, 2019, Iivanainen, Zetter, Grön, Hakkarainen, Parkkonen, 2019). The practical use of these solutions is therefore hardware demanding and expensive, partially reducing the benefits brought by the use of ”zero-field” sensors.

In this work we take a complementary approach: we realize and use for MEG an OPM sensor that is resilient to non-compensated magnetic fields and does not require additional peripheral hardware. Our OPM sensor is based on the non-linear magneto-optical rotation (NMOR) technique (Budker, Kimball, Yashchuk, Zolotorev, 2002, Kimball, Pustelny, Yashchuk, Budker, 2013, Pustelny, Wojciechowski, Gring, Kotyrba, Zachorowski, Gawlik, 2008). We demonstrate the detection of auditory evoked fields in an environment with background magnetic field of 70 nT. We benchmark our sensor with a commercial SQUID sensor, also demonstrating the possibility for measuring stimulus evoked modulations in oscillatory brain activity. In comparison to the zero-field magnetometers employed so far for MEG, the main advantages of our sensor are the higher dynamic range and the enhanced resilience to external magnetic field fluctuations. This effectively reduces the requirement for shielding and active field compensation. Additionally, the NMOR does not require coils to modulate or null the field, therefore removing cross-talk problems.

**Fig. 1:**
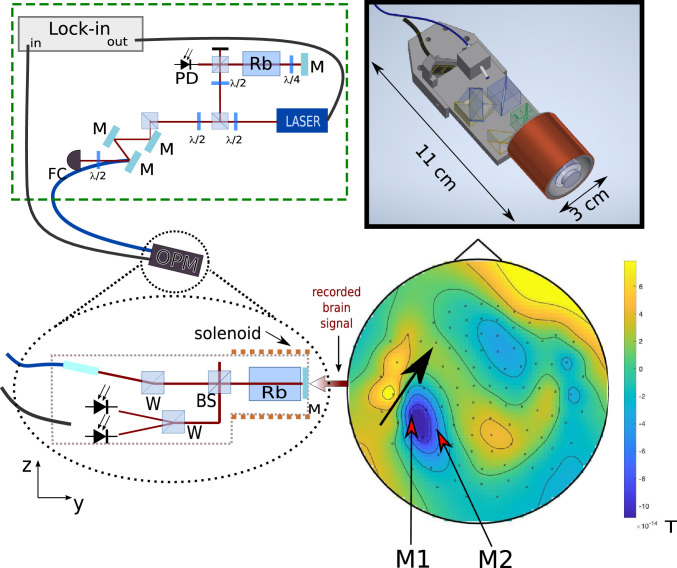
Schematic representation (not to scale) of the NPOM modular system. The external module (the dashed box) includes a laser and the optics for fiber coupling, the electronics (not shown) and the lock-in amplifier. The output of the lock-in amplifier is used to modulate the frequency of the laser. M indicates the mirrors, PD the photodiodes and FC the fiber coupling. The external module is connected to the sensor head through an umbilical that contains an optical fiber that delivers the light (blue line), a shielded coaxial cable that carries the output of the sensor and a wire to set the bias field in the sensor (black line). BS indicates the 90/10 beam splitter and W the Wollaston prisms. When placed as shown, the sensor measures the component of the magnetic brain signal that is perpendicular to the participant’s head. The contour map illustrates the auditory evoked response measured at 100 ms by the whole-head SQUID system (magnetometers). The black arrow indicates the radiating dipole. The inset on the top right shows a 3D rendering of the sensor head.

When building the sensor, we chose a modular approach, such that the sensor head includes only non-magnetizable parts, and the laser and electronics are connected via optical fibers and shielded cables (Fig. 1). Such an arrangement effectively reduces the magnetic artifacts from the electronics, but also enables more versatile designs compared with integrated sensors that are currently commercially available. For instance, a single laser can drive several sensor heads, and our technique can be combined with transcranial magnetic stimulation (TMS), whose high magnetic field pulses could damage the laser and electronic components integrated in the sensor heads. Our approach holds the promise of combining brain stimulation with OPM recordings in order to estimate task-dependent functional connectivity between brain regions.

## 2 Methods

Our nonlinear OPM (NOPM) is based on the NMOR effect, which has been known since the 1960s and exploits polarized light nearly resonant with an atomic transition. When the light propagates through an atomic gas, it optically pumps the atoms in a state that is aligned with the polarization of the light. In the presence of an external magnetic field, the atomic alignment rotates around the direction of the magnetic field at the Larmor frequency $\Omega_{L}=g\mu_{B}B/\hbar ,$ where B is the magnitude of the external magnetic field, $\mu_{B}$ the Bohr magneton, g the Landé g-factor and ℏ the reduced Planck constant. This, in turn, rotates the polarization of the light. The frequency of such rotation is proportional to the magnitude of the magnetic field and can therefore be used to measure it (Bell, Bloom, 1961, Optical Magnetometry, Kimball, Pustelny, Yashchuk, Budker, 2013).

To promote the nonlinear effect, a synchronous stroboscopic optical pumping is applied. For linearly polarized light, if the pumping modulation rate is twice $\Omega_{L},$ the medium is *resonantly* pumped into the aligned state that rotates around the magnetic field. The resonance condition can be tuned by modulating the laser amplitude or frequency. Determination of the resonance frequency can be used to perform precise magnetometry as long as the bias field lies within the dynamic range, which for NMOR can be extended up to geomagnetic fields (Acosta et al., 2006). The theoretical sensitivity limit of the NMOR technique is lower than 1 fT$/\sqrt{\text{Hz}}$^1^.

### 2.1 The NOPM sensor

In our sensor we induce the NMOR effect by modulating the frequency of the light (FM NMOR) (Budker et al., 2002). In Fig. 1 we show a schematic representation of our system. We have adopted a modular approach in which all electronic and magnetic components are kept outside the magnetically shielded room (MSR) in which we perform the human recordings. A 5 m umbilical cable connects the external module with the sensor. The umbilical combines an optical fiber, which delivers the light to the sensor, a shielded coaxial cable, which brings the sensor output outside the MSR, and a copper wire that delivers DC currents to the sensor head.

In the external module outside the MSR, the laser light is provided by a Toptica DL Pro diode laser at 795 nm. The frequency of the laser is red-detuned by ≃480 MHz with respect to the $\left|F=2\rangle \to \right|F^{\prime }=1\rangle$ hyperfine transition of the ${}^{87}$ Rb D1 line. To excite the NMOR resonance, we implement the frequency modulation by directly modulating the injection current of the laser diode. The sinusoidal modulation signal, at frequency $\Omega_{m},$ is generated by a lock-in amplifier (Zurich Instruments MFLI) and the typical amplitude of the modulation is ≃1 GHz.

The output of the laser passes through an optical isolator and part of it is sent to a saturated absorption spectroscopy setup, for frequency referencing. The rest of the light, suitably attenuated, is delivered to the sensor head using a 5 m long polarization maintaining fiber.

#### 2.1.1 The sensor head

A schematic representation of the sensor head is given in Fig. 1. Inside the sensor head, we use a single beam both to pump and probe the atoms. The beam delivered by the fiber is collimated to have 0.5 mm beam waist using a pigtailed GRIN lens glass collimator. The light polarization is further cleaned using a Wollaston prism and sent through a 90/10 non-polarizing beam splitting cube. 90% of the incident power is reflected and sent to a photodiode, whose output can be used in a feedback loop to compensate for polarization fluctuations in the fiber. The remaining 10% is sent to the atomic vapour cell, which has antirelaxation paraffin coated walls. The use of a paraffin coated cell allows us to work at room temperature^2^. The cell is a 2 cm long cylinder with 1.8 cm diameter base and is placed inside 3 cm diameter solenoid that provides a constant bias magnetic field along the radial y direction (see Fig. 1).

The bias field is used to control the ‘carrier’ Larmor frequency $\Omega_{L}^{0}$ at which the atomic alignment rotates in the absence of any brain signal. To improve the resilience of the sensor, the bias field should be significantly larger than any background field. This ensures that $\Omega_{L}^{0}$ is as stable as possible and minimally perturbed by the background field. Indeed, every component of the background field that is not parallel to the bias field is strongly suppressed. Our sensor is therefore designed to measure magnetic fields along the y direction, that cause tiny variations δΩ around $\Omega_{L}^{0}$. In our working conditions, the bias field is typically in the range of 100-150 nT, and is generated using an ultra-low noise current supply (HighFinesse BCS 20 mA). This bias field overcomes the residual field inside the MSR, which is typically between 40 and 100 nT (see below).

After passing the cell, the beam is retro-reflected on a 1 mm thin mirror with 0${}^{\circ }$ incidence angle. This not only ensures that the gap between the subjects scalp and the vapour cell is minimized, but also effectively doubles the atom-light interaction length. For the measurements presented, the end of the sensor that contains the reflecting mirror was placed 2 mm from the scalp. As depicted in Fig. 1, our sensor measures the component of the magnetic brain signals that is radial to the brain.

On the way back, the beam passes again the 90/10 cube and the rotation angle is analysed using a balanced polarimeter. This comprises a Wollaston prism mounted at 45${}^{\circ }$ with respect to the incident light polarization and two low noise photodiodes in a ceramic assembly (Hamamatsu S1337-33BQ) connected in series. In this configuration the photocurrents cancel each other when they are equal. Including all these elements, the current sensor head is a cylinder with base area of 3 cm diameter and length of 11cm. By using smaller cells and miniaturized optics, it is possible to further reduce the dimensions of the sensing head without compromising the performance.

The output of the polarimeter is sent with a 5 m long SMA cable to a transimpedance amplifier (Koheron PD01). This amplifies the signal by a factor of $10^{5}$ and minimizes dark currents by operating the photodiodes with no bias voltage. The signal is then digitized using the analog-to-digital converter built in the lock-in amplifier, featuring 16 bit resolution and 60 MSa/s conversion rate. The digitized signal is then demodulated at $\Omega_{m}$.

### 2.2 Operating the sensor

We operate the sensor in two modes, *unlocked* and *tracking*. In the unlocked mode, we obtain a dispersive and an absorptive curve at the in-phase and quadrature outputs of the lock-in amplifier, respectively. We use the unlocked mode to optimize the NOPM performance and choose the optimal working conditions. As mentioned above, the measurement of the magnetic field is performed by measuring the frequency of the NMOR resonance. In order to fast-track the changes, we operate the magnetometer in the tracking mode. In this mode, the signal provided by the balanced photo-diode is fed directly to the laser current modulator to realize a phase locked loop (PLL). The PLL has programmable center frequency and phase set point. We determine the input parameters by sweeping $\Omega_{m}$ around the resonance in the unlocked mode. If the environmental conditions change we readjust these 2 parameters and re-lock the magnetometer. The output parameter of the PLL is the oscillator frequency, which we record together with the frequency shift using the digital acquisition (DAQ) module incorporated in the lock-in amplifier^3^. Any change in the magnetic field due to brain activity is immediately sensed by the atoms and reflected in a change of the modulation frequency. In other words, the atoms themselves set the frequency determined by the magnetic field in real time (Bloom, 1962). Therefore tracking the magnetic field is realized by precise frequency counting. In the tracking mode, our sensor can have up to 1 kHz bandwidth.

We have tested our sensor to work at least up to 1 µT external magnetic fields. In principle the NMOR sensor can operate with bias fields comparable with geomagnetic fields (Acosta et al., 2006), but at the cost of a lower sensitivity. Indeed, high magnetic fields usually imply high magnetic field noise and magnetic field gradients. For the measurement presented in this work, we have chosen $\Omega_{m}=2\Omega_{L}^{0}=$1.5 kHz, which corresponds to a 110 nT bias field, as this is few times higher than the residual magnetic field in the MSR at the position of the participant’s head, and allows a competitive sensitivity. We found that we achieve our best performance when the power of the beam is set to 7 μW in front of the cell.

The overall performance of a system is determined by the combination of the intrinsic sensitivity of the sensor and the characteristics of the external magnetic field. Our MSR is made of 2 layers of μ-metal and 1 layer of aluminium (Vacuum Schmelze) with the residual magnetic field measured upon installation to be ∼4 nT. The room is built for conventional MEG experiments and has an MEGIN TRIUX system installed inside, together with a cryocooler. The cryocooler cold-head makes for a challenging environment as it produces spurious magnetic fields and magnetic field gradients inside the MSR Meg. At 2 m distance, where we operate our NOPM, the background magnetic field is ≥70 nT and the magnetic field gradient can be up to 240 nT/m. Zero-field OPM sensors are not able to work in such environment without extra compensation system (Holmes, Tierney, Leggett, Boto, Mellor, Roberts, Hill, Shah, Barnes, Brookes, Bowtell, 2019, Iivanainen, Zetter, Parkkonen, 2019). Crucially, our NOPM sensor is far less affected, as it employs a bias magnetic field. As explained above, for sufficiently large bias fields, NMOR sensors are insensitive to transverse gradients (Pustelny et al., 2006). Therefore, we oriented our sensor to have the smallest gradients in the direction of the measurement. Any variation of the cryocooler field, mostly due to vibrations, can additionally affect OPM sensors placed inside the room. In our MSR, we have measured slow variations from 50 pT to 1 nT per hour, and sudden jumps of up to 3 nT. In case of sudden jumps larger than the resonance width of 20 Hz (1.5 nT) the sensor unlocks. These are rare events, and the sensor can be readily re-locked without any further calibration. The dynamic range of the sensor is quite large and we measured slow drifts with amplitude up to 100 nT. The ability to operate in high, inhomogeneous and varying fields is a crucial asset of our sensor.

### 2.3 Participant and experimental paradigm

A healthy male subject aged 35, with normal hearing and no history of neurological or psychiatric disorders was recruited. The experiments took place at the Centre for Human Brain Health at University of Birmingham, United Kingdom. The research protocol was approved by the the Science, Technology, Engineering and Mathematics Ethical Review Committee at the University of Birmingham. The participant was informed about the experimental procedure during the SQUID and NOPM experiments and written consent forms for both sessions, which took place on different days, were obtained.

We recorded auditory ERFs in two sessions: one using a conventional SQUID-based MEG system and one using our NOPM. During the SQUID session, signals were continuously recorded using a 306 sensor MEGIN TRIUX system. During the NOPM session a single sensor OPM recording was obtained

During both sessions, the subject was presented with two 100 ms tones of respectively 1 kHz and 1.04 kHz and the inter-stimulus interval was randomly varied from 811 to 840 ms. The sound was generated using a SOUNDPixx MRI compatible audio system and was delivered to the right ear of the participant using air tubes and disposable earpieces. The subject was not required to respond but was asked to concentrate on the tones throughout the experiment and was instructed to remain still for the duration of the experiment. In the SQUID session we recorded a total of 300 trials. During the SQUID session the subject was seated while in the NOPM session he was lying in the supine position, with the head comfortably supported. For the NOPM measurements the recording area was identified using the data obtained in the SQUID session. We identified the MEGIN-MEG sensors with the strongest magnetic field response and placed the NOPM sensor approximately in the same location. The sensor was fixed to the bed using non-magnetic materials. We recorded a total of 450 traces in the NOPM session.

### 2.4 Data acquisition

For both the SQUID and the NOPM the duration of each trial was about 1 s. Each trace included a 100 ms pre-stimulus recording, which we use for baseline estimation.

The data of the SQUID session were acquired with a 306 channel whole-head MEGIN TRIUX system. The data were filtered by a 330 Hz low-pass and 0.1 Hz high-pass filters and then sampled at 1 kHz.

The data of the NOPM session were recorded using the DAQ module integrated in the lock-in amplifier and down-sampled to 1.35 kSa/s (from 60 MSa/s). The length of each trace was set to 970 ms.

### 2.5 Data analysis

One of the main challenges of operating a single OPM sensor in the MSR is to deal with the drifts of the background magnetic field. To minimize the effect of such drifts, we removed traces where the external magnetic field drifted more than 1.5 pT per second, resulting in 200 usable trials. This was done because such drifts exceed the expected magnitude of the brain signal. The auditory event-related fields were calculated by applying a 35 Hz low-pass filter, averaging the trials, and subtracting the 100 ms baseline interval.

For the SQUID session, we selected for comparison the magnetometer that measured the strongest ERF (labelled in the following as M1) and the magnetometer that provided the most similar response to the ERF (labelled as M2). Using the same criterion as the NOPM session to minimize the effect of drifts in the magnetic field i.e., removing the traces with drifts higher than 1.5 pT/s, we obtained 200 trials from the SQUIDs sensors. The traces were low-pass filtered at 35 Hz and then averaged. A 100 ms baseline was subtracted. We did not apply MaxFilter or signal-space projections to the M1 sensor for a fair comparison of the performance with the NOPM data. Conversely, such techniques were applied to the M2 sensor to better highlight the shape of the auditory evoked response.

For both sessions, the time-frequency representation of power was calculated per trial using a 300 ms time-window sliding with 50 ms time steps. A Hanning taper was applied to each 300 ms time-window prior to Fast-Fourier Transformation, after which the power was derived.

## 3 Results

Our core aim was to record auditory evoked fields from a human participant, demonstrating that our NOPM sensor can be used for MEG in a shielded room without active field compensation.

**Fig. 2:**
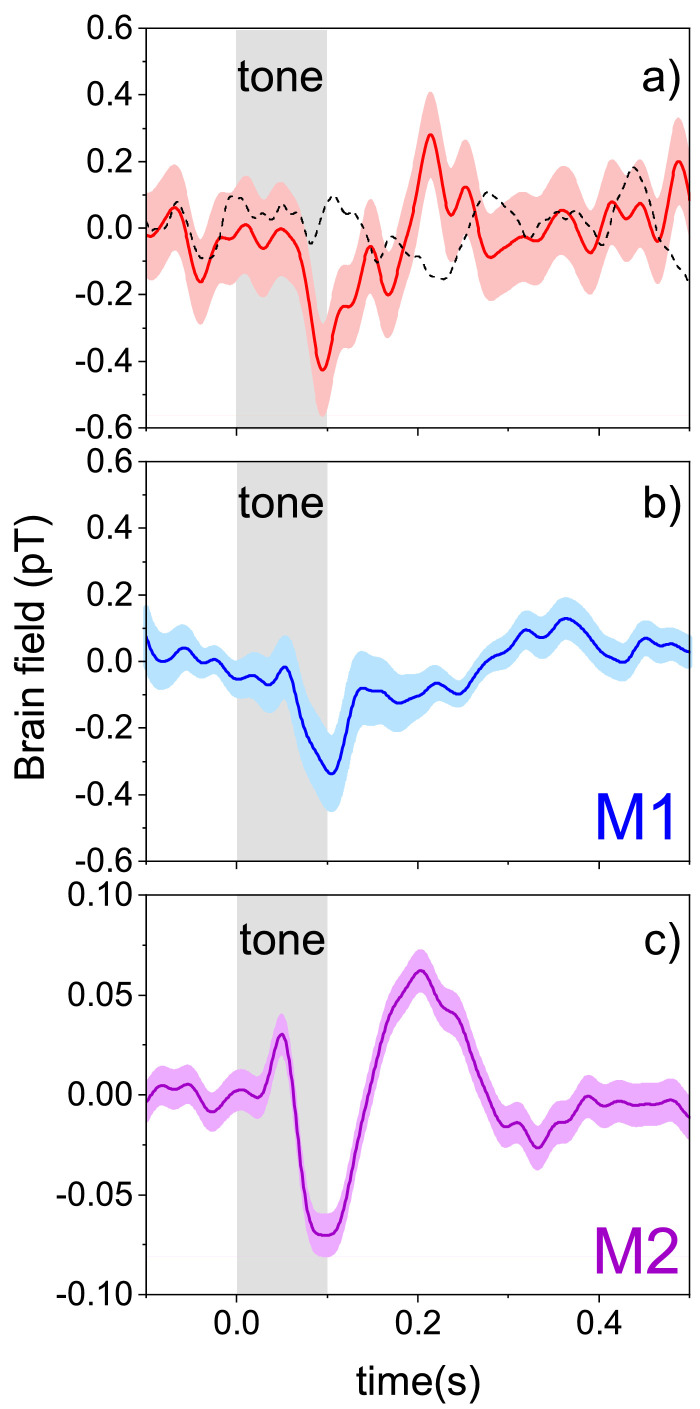
a)The auditory event-related field (ERF) measured by the NOPM sensor averaged over 200 trials. Note the strong brain response at ≃100 ms (the response is significantly larger at 100 ms than the baseline interval; paired t-test; p=0.002) and at ≃200 ms (also paired t-test, p=0.002). The shaded area indicates the standard error of the mean. For comparison we report the trace measured during ‘dummy’ trials (dashed black line), where no tone was presented to the participant. b) Representative ERF trace of the Triux MEG system. This sensor (M1) was chosen for comparison as it provided the strongest response to the ERF. c) A trace from another sensor (M2) of the Triux MEG system chosen for the similitude with the NOPM response. In this case MaxFilter and signal-space projections were additionally applied to better highlight the shape of the auditory evoked response. Note the vertical scale smaller by a factor of 3, as the amplitude of the signal is lower than in the other panels.

Fig. 2 shows the event-related field in response to ∼200 tones for the NOPM and the SQUID sensors respectively. In all the three panels, we observe a strong deflection at ≃100 ms which correspond to the N100m (Hämäläinen et al., 1993). We performed a paired *t*-test for the NOPM by comparing the response at 100 ms versus the baseline, confirming a highly significant response (p=0.002). The NOPM response is slightly stronger than the M1 response and significantly stronger than that of the M2: 427 fT against 337 fT and 70 fT respectively. This is due to the closer proximity to the scalp. The noise level of the NOPM traces is higher: the average standard error of the mean for the NOPM is ≃130 fT, while for the M1 and M2 SQUIDs it is ≃60 fT and ≃11 fT respectively. Note however that for M2 this performance is obtained using advanced filtering, as explained above, so it cannot be directly compared. The higher noise level in the NOPM recordings is mainly due to magnetic field noise coming from the vibrations in relation to the MEGIN-MEG cryocooler. The SQUID sensors are not affected by such noise as they are rigidly connected to the cryocooler. By comparing the signal-to-noise ratio for these traces, i.e., the ratio between the amplitude of the N100m peak and the average standard error of the mean reported above, we obtain ≃3.3 for the NOPM sensor and ≃5.6 for M1 SQUID sensor. Therefore our prototype NOPM sensor delivers performance slightly lower but in the same range of those of state-of-the-art SQUIDs. The NOPM trace presents a peak at 200 ms, which is also highly significant compared to the baseline according to a paired t-test. Similar peak is observed in the M2 trace.

**Fig. 3:**
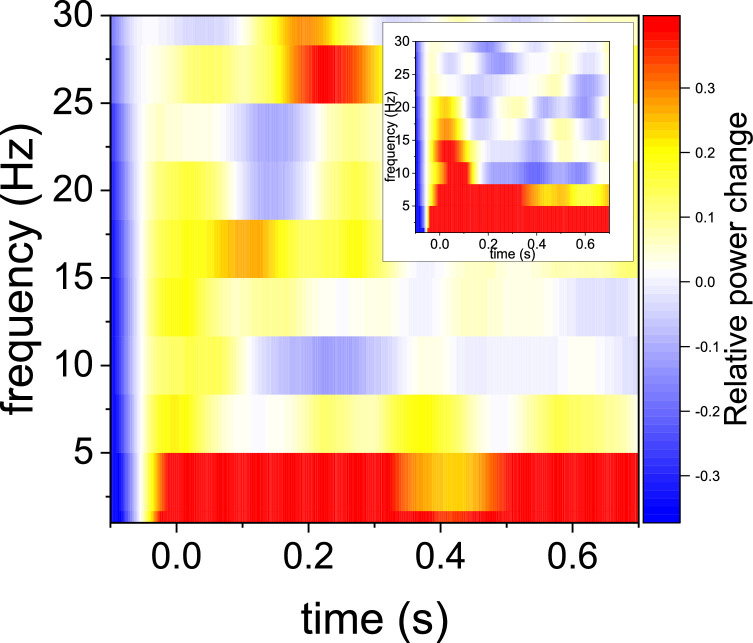
Time-frequency representation of power measured by the NOPM sensor in response to the tone. The color scale indicates the relative power modulation. The power estimates were calculated per trial and then averaged. The response is relative to the baseline. Note the typical depression in alpha-band power at around 10 Hz in response to the tone. The inset shows for comparison the same time-frequency representation for the M2 SQUID sensor.

To further investigate the response, we calculated the time-frequency representation of power for each trial of the NOPM session (see Methods). These power estimates were then averaged. The results are reported in Fig. 3, and reveal the expected robust depression with respect to the baseline in the alpha band around 10 Hz, a few hundred ms after the tone (t-test for the power for the 10 Hz band in the full 0.15–0.7 s interval, *p*<0.05).

## 4 Discussion

With only one sensor it is obviously not possible to decouple the environmental contribution from the intrinsic sensitivity of the sensor itself. To estimate the actual sensitivity in our working conditions, i.e., the combined contribution of the intrinsic sensitivity of the sensor and the environmental noise, we recorded the width w of our resonances in the unlocked mode, and we measured the signal-to-noise ratio S/N from the square root of the power density spectrum. From this, the actual sensitivity can be evaluated as Pustelny et al. (2008):

(1) $$\sigma_{a}=\frac{\pi \hbar }{g\mu_{B}}\frac{w}{S/N}.$$

**Fig. 4:**
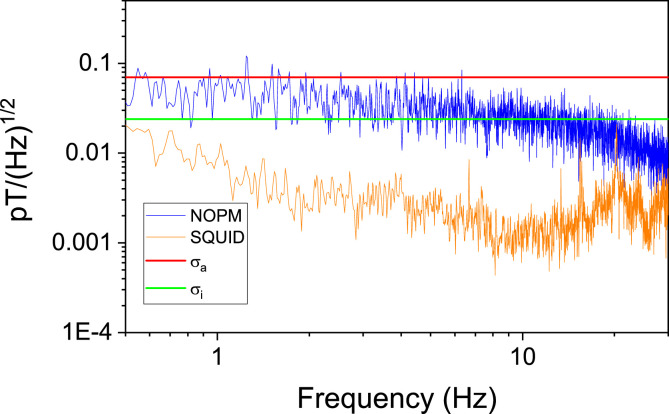
Uncorrected noise spectrum of ‘empty-room’ recordings for the NOPM and SQUID sensors in the frequency range relevant for this work. The horizontal lines are the calculated actual and intrinsic sensitivities of the NOPM sensor $\sigma_{a}$ and $\sigma_{i}$. For the NOPM, the major noise source at low frequencies is due to the environmental noise that were better controlled for the SQUIDs than the NOPM.

For our setup, the typical value of w is 20–25 Hz, while the S/N is ≃20,000, yielding a sensitivity $\sigma_{a}≃$70 fT$/\sqrt{\text{Hz}}$. This value is in excellent agreement with the limit set by the measured noise spectrum, as shown in Fig. 4. In the same figure we report for comparison the noise spectrum measured with the SQUID magnetometer of Fig. 2c.

The physics of NMOR magnetometers is very well known and, from the same measurement, it is possible to estimate the intrinsic sensitivity $\sigma_{i}$ of our NOPM sensor, which is essentially given by the photon shot noise. This can be evaluated as

(2) $$\sigma_{i}=\frac{\pi \hbar }{2g\mu_{B}}\frac{w}{A}\sqrt{\frac{1}{n}},$$

with A the amplitude of the NMOR signal and n the number of photons in the beam per unit of time. For our working conditions this yields $\sigma_{i}≃$24 fT$/\sqrt{\text{Hz}}$. This can be further improved using a lower bias field (at 15 nT the width of our resonance is 25% smaller) and higher power, reaching intrinsic sensitivities comparable with commercially available zero-field sensors. Knowing the value of $\sigma_{i}$ from Fig. 4, it is possible to appreciate that the environmental noise is the dominating contribution to $\sigma_{a}$. In our case the cryocooler was the major noise source. We would therefore expect a much better actual sensitivity in a dedicated MSR.

In conclusion, we have implemented a modular OPM sensor based on the NMOR technique, in which the sensor head contains no elements that can be magnetized. The electronics, as well as the laser source are kept in an external module, placed outside the MSR. We have employed the sensor to detect the auditory evoked fields from a human participant, and compared it with state-of-the-art commercial SQUID magnetometers from the MEGIN system. We report comparable performance for our NOPM sensor, especially considering that the SQUID sensors are not affected by the noise of the cryocooler. We speculate that, in a dedicated (empty) MSR, our sensor would outperform the SQUIDs given the closer proximity to the scalp. Furthermore, spectral analyses confirmed that our NOPM sensor is able to detect other typical features associated with brain response, such as the reduction of the ≃10 Hz alpha band activity.

Our NOPM sensor provides a complementary approach to OPM-based MEG with respect to zero field sensors. Our sensor can be used in environments with non-compensated magnetic fields, greatly simplifying the peripheral hardware requirements such as compensation coils. The *actual* sensitivity, resulting from the combination of the intrinsic sensitivity of the sensor and the uncompensated environment, is sufficient to reliably detect the auditory evoked response. The *intrinsic* sensitivity is instead slightly higher but of the same order of magnitude of commercial zero-field sensors.

In the future, we will assess the performance of our sensor on moving participants. Indeed, the ability to perform MEG recordings during movement is one of the most exciting features of OPM systems (Boto et al., 2018). Our sensor could potentially present advantages in this respect, perhaps easing the requirement for the stabilization of the background field. Due to the use of the FM NMOR technique, our sensors will have no cross-talk interference, making them particularly appealing for multi-sensors detection arrays 4. Arranging multiple NOPM sensors in configurations similar to the ones used in SQUID-based MEG systems, in particular exploiting the gradiometer setup (Zhang et al., 2020), could further improve the resilience of these sensors to external magnetic field fluctuations. Exploiting its modular feature, our NOPM sensor could represent a cost-effective versatile solution for cognitive and clinical neuroscience applications. For example, many sensors could be driven by a single laser source, and the absence of electronic components in the sensor head makes it compatible with TMS, opening up new possibilities in the detection and treatment of the human brain.

## CRediT authorship contribution statement

**Anna U. Kowalczyk:** Conceptualization, Methodology, Investigation, Formal analysis, Data curation, Writing - original draft, Funding acquisition. **Yulia Bezsudnova:** Methodology, Investigation, Formal analysis, Data curation. **Ole Jensen:** Conceptualization, Methodology, Formal analysis, Writing - review & editing, Supervision, Funding acquisition. **Giovanni Barontini:** Conceptualization, Methodology, Investigation, Formal analysis, Writing - review & editing, Supervision, Funding acquisition.

## Declaration of Competing Interest

None.

## Footnotes

1 Such sensitivities can be achieved using NMOR with atomic density 10${}^{12}$ atoms/cm${}^{3}$ and light intensity 2 mW/cm${}^{2}$ (Budker, Kimball, Yashchuk, Zolotorev, 2002, Fleischhauer, Matsko, Scully, 2000).

2 The paraffin coating allows the atoms to collide with the walls around 10000 times without losing polarization. Increasing the temperature can increase the signal amplitude up to a factor of 2 at the maximum temperature of 50° (Pustelny et al., 2008).

3 Note that for data analysis presented here we are using the frequency shift from the magnetometer lock-point rather than the absolute $\Omega_{m}$ as the lock-in amplifier is able to measure it with better precision.

4 The only effect coming from placing two NOPM sensors close is a static shift of the bias field $B_{0}^{\prime }=B_{0}-B^{\prime }$. Considering two NOPM sensors identical to the one presented in this work placed at a distance of 2 cm, we have that $B^{\prime }≃2AB_{0}/\left(\pi L\right),$ with A and L the base area and length of the solenoid producing the bias field. For our specific case where $B_{0}=110$ nT, we have that $B^{\prime }≃20$ nT and therefore $B_{0}^{\prime }≃90$ nT. As the value of the ’carrier frequency’ does not bear any information, such shift has no effect on the performance of the sensor. The shift is further reduced if the sensors are not parallel or further apart.
